# 钝性伤致下肢血管断裂及创伤性凝血病综合治疗体会

**DOI:** 10.3760/cma.j.cn121090-20241225-00589

**Published:** 2024-12

**Authors:** 向阳 李, 兰娟 徐

**Affiliations:** 郑州大学附属郑州中心医院重症医学科，郑州 450001 Department of Critical Care Medicine, Zhengzhou Central Hospital, Zhengzhou University, Zhengzhou 450001, China

## Abstract

临床中创伤导致的血管损伤并不少见，可能导致创伤性凝血病、肢体缺血性坏死，可导致截肢或因失血性休克而死亡，与不良结局明显相关。回顾性分析我科1例53岁男性严重撞击伤患者，挤压伤导致下肢股动脉离断、下肢缺血及创伤性凝血病，利用输血器制作转流器急诊行血管临时转流，期间经历凝血功能紊乱、横纹肌溶解、骨筋膜室综合征以及创面感染，通过补充凝血底物纠正凝血功能、加强抗感染、切口减张肌肉及多次清创手术，从而保住一侧肢体的诊疗过程。对于创伤性血管损伤，尤其是钝性损伤，早发现、早干预、早治疗，及时调整手术策略，从而避免肢体缺血、坏死，避免不良结局。

## Abstract (en)

Vascular injury caused by trauma is not uncommon in clinic, which may lead to traumatic coagulopathy, ischemic necrosis of limbs, amputation or death due to hemorrhagic shock, which is obviously related to adverse outcomes. A 53-year-old male patient with severe impact injury in our department was retrospectively analyzed. Crushing injury resulted in femoral artery disconnection of lower limbs, which led to ischemia and traumatic coagulopathy of lower limbs. A blood transfusion device was used to make a shunt for emergency temporary vascular bypass, during which he experienced coagulation disorder, rhabdomyolysis, osteofascial compartment syndrome and wound infection. By supplementing coagulation substrate, correcting coagulation function, strengthening anti-infection, reducing muscle tension through incision and multiple debridement operations, the diagnosis and treatment process of one limb was preserved. For traumatic vascular injuries, especially blunt injuries, early detection, early intervention and early treatment, and timely adjustment of surgical strategies can avoid limb ischemia and necrosis and reduce adverse outcomes.

严重创伤是一个重大的全球公共卫生负担。全球疾病负担研究表明，2019年全球9％死亡与创伤有关，血管损伤是导致高死亡率及致残率的主要原因[1]。半数患者在创伤后2 h内因失血而死亡，且创伤后24 h内死亡的绝大多数为大量失血患者，25％～35％的严重创伤患者入院时出现创伤性凝血病（TIC）[1]。TIC是创伤后由组织损伤引起的凝血功能障碍，其相关凝血异常可表现为高凝或低凝，临床症状和体征可相应表现为血栓栓塞或难以控制的大出血。临床中外伤导致的血管损伤并不少见，超过80％的动脉损伤发生在下肢，可同时合并下肢静脉、骨骼、神经、肌肉损伤，若未及时诊断或治疗不当，可能导致患者因下肢缺血性坏死而截肢，甚至出现失血性休克而死亡。因此对于下肢损伤患者，早期识别及救治至关重要，本文报道了1例严重挤压伤合并血管断裂患者的诊治过程，并通过文献回顾来探讨血管断裂患者早期识别及诊治方法。

## 病例资料

患者，男性，53岁，身高175 cm，体重80 kg（BMI为26.1）。以“重物撞击致右腹股沟肿胀、疼痛5 h”入院。既往长期抽烟20余年，每日约20支。无其他基础病病史，无食物药物过敏史，有输血史，无家族史，患者于5 h前锯木时被长约2 m、直径40 cm木料弹回撞击右侧腹股沟区，随即出现右侧腹股沟区肿胀，伴青紫，无开放性伤口，骨擦音及骨擦感；自行按压受伤部位，家人急送至当地医院，给予输血、补液等对症治疗（具体不详），逐渐出现下肢冰凉、麻木、无力、肢体活动障碍等，为求进一步治疗，遂转至我院。入院按照创伤CRASHLAN查体：C：心脏相对浊音界正常，心率105次/min，律齐，各瓣膜听诊区未闻及明显杂音，无心包摩擦音，无周围血管征；R：双侧胸廓对称，听诊双肺呼吸音稍粗，未闻及干啰音，无胸膜摩擦音；A：腹肌紧张，右下腹压痛、无反跳痛，墨菲征阴性，肝区无叩痛，肾区无叩击痛，肠鸣音稍弱，每分钟2次，无气过水声；S：脊柱生理弯曲存在，无畸形，无明显压痛及叩击痛；H：头颅大小正常无畸形，结膜稍苍白，巩膜无黄染，双侧瞳孔等大等圆，直径约2.5 mm，对光反射灵敏；P：骨盆挤压试验及分离试验阴性；L：右侧腹股沟区可见直径约20 cm肿胀青紫，右下肢肿胀，颜色青紫，皮温凉，感觉及运动功能缺失，皮肤温度低；A：右足背动脉均未触及；N：神志清，精神稍差，颈软，无强直，左侧肢体活动可，左侧巴宾斯基征阴性，右侧未引出。完善急性双下肢CTA、胸腹部CT等检查，以“下肢损伤”为诊断收入我科，胸部+腹部+骨盆CT提示：①右侧腰大肌、髂腰肌、腹肌及右侧骨盆诸肌群软组织肿胀，密度增高，考虑血肿形成；②右侧腹腔、盆腔积血积液，右侧阴囊内血肿形成；③右侧髂骨棘骨折；④双肺弥漫性小叶中心结节，考虑呼吸性细支气管炎；⑤双肺下叶少许条索灶；⑥主动脉及冠状动脉硬化。双下肢CTA提示：①盆腔右侧及右侧股骨上段巨大血肿，右侧髂外动脉受压，髂外动脉局部管腔可疑充盈缺损（不除外血栓形成可能），右侧髂外动脉远端管腔、右下肢动脉管腔全程未见显示，考虑闭塞。②腹主动脉及左下肢动脉硬化；腹主动脉、双侧髂总、髂内、左侧髂外动脉局部轻度狭窄；左侧股动脉、腘动脉软斑、混合斑，管腔轻度狭窄；左侧胫前动脉软斑、钙斑，远端管腔未显影；左侧胫后动脉及腓动脉管腔纤细，远端未见显影。③右下肢软组织弥漫性肿胀。结合患者右大腿局部肿胀、足背动脉波动消失及CTA结果，经我院创伤多学科团队评估后考虑为钝性伤导致下肢血管断裂，外周介入科、骨科、血管外科、普外科联合会诊后，急诊由介入科行“腹主动脉造影+双侧髂动脉造影+下腔滤器置入术”，随后血管外科行“双下肢血管探查+股-股临时血管转流（TIVS）+筋膜组织瓣成形术”，因手术紧急，无专用人工血管，将输血器临时制成转流器行血管吻合，术中探查血流畅通，术后转入ICU综合治疗。

入科时神志清，生命体征稳定［体温36.0°C，心率95次/min，呼吸20次/min，血压：116/71 mmHg（1 mmHg=0.133 kPa）］。入科实验室检查：pH 7.25，碱剩余−9.4 mmol/L，白细胞计数10.78×10^9^/L，血红蛋白78 g/L，血小板计数97×10^9^/L；凝血四项：凝血酶原时间（PT）23.1 s，活化部分凝血活酶时间（APTT）>150 s，纤维蛋白原（FIB）1.32 g，国际标准化比值（INR）2.03，D-二聚体12.98 mg/L；肌红蛋白：31 302 ng/ml；凝血及血小板功能分析：凝血酶生成时间553 s，血小板功能0.41，凝血速率3.5 min^−1^，余肝肾功能及脏器功能尚可，存在诊断为创伤性凝血病，早期打破凝血功能障碍、低体温及酸中毒“死亡三角”，治疗上立即建立中心静脉通路，启动紧急输血流程，肢体保暖、输血输液加温装置、碳酸氢钠应用纠酸，输注纤维蛋白原4g、凝血酶原复合物1 200 U，同时输注悬浮红细胞2 U、冰冻血浆800 ml、10 U冷沉淀，氨甲环酸（1 g静脉输液同时1 g静脉泵入维持8 h）抗纤溶，哌拉西林他唑巴坦抗感染，患者循环体征趋于稳定，监测下肢动脉超声血流通畅，下肢皮温及颜色较前改善。

伤后第2天上午右下肢明显肿胀，考虑骨筋膜室综合征，骨科急诊在床旁行右侧大腿+小腿筋膜间室切开减张术，沿右侧大腿外侧行一约30 cm、右侧小腿外侧行一约25 cm、右侧小腿内侧行一约25 cm大小的纵向切口，术中可见肌肉4C征，右侧大腿张力明显减低，右侧小腿张力明显减低，当日下午血管外科再次行“股动脉-股动脉人工血管搭桥术+腹主动脉造影术+双侧髂动脉造影术”，术中腹主动脉未见明确异常、腹腔干、双肾动脉、肠系膜上下动脉未见明确异常。

伤后第3天，患者右下肢肿胀再次加重，同时减张切口处持续渗血严重，紧急床旁电凝刀彻底止血，监测凝血功能提示PT 14.7 s，INR 1.29，APTT 78 s，活化凝血时间（ACT）277 s，再次输注4 g纤维蛋白原、1 200 U凝血酶原复合物、10 U冷沉淀、600 ml冰冻血浆，1治疗量血小板以及4 U悬浮红细胞等凝血底物及血制品，纠正凝血功能紊乱，患者入科即少尿，炎症指标升高明显，持续血液净化，肌红蛋白最高值31 302 ng/ml，结合病史考虑横纹肌溶解综合征，立即高通量炎症因子吸附，随后应用枸橼酸抗凝持续血滤纠正肾功能。

经过调整抗感染、改善循环、早期抗凝、营养支持等治疗措施，患者体征趋于稳定，伤后12 d转入骨科继续治疗，期间多次行“右大腿、小腿骨筋膜室综合征切开减张术后扩创术+带蒂复合组织瓣成形术+负压吸引术”，伤后108 d转入显微外科继续治疗，总住院时长300余天，期间共行10次“皮肤扩创+植皮+筋膜成形手术+血管成形术”，患者最终恢复良好，遗留右下肢轻度跛行。

## 讨论

创伤后出血是导致患者死亡的原因，开放性血管损伤常伴有出血或缺血表现，较容易发现，而闭合性损伤病情隐匿，临床表现更为复杂，常于术中探查或因术后迟发性缺血而被发现[2]。由于漏诊和延迟治疗等问题，钝性血管创伤与较高的截肢率和病死率有关，因为高能量转移会导致广泛的组织破坏[3]。动脉或静脉的损伤可发生在骨折时，术中或术后，甚至发生于伤后数周、数月甚至数年[4]。

创伤性凝血病仍然是创伤死亡的主要原因之一，发生率高达25％，致死率为30％～50％[5]。据报道，创伤后无法控制的出血部分是由创伤引起的创伤性凝血病，是机体组织损伤和失血的内源性反应过程，因此在严重创伤患者早期，血制品、凝血底物的补充至关重要。与此同时，血管的持续出血是凝血功能紊乱的因素之一，如果不及早控制出血和进行适当的复苏，患者凝血因子耗尽，同时合并复苏后稀释及创伤的破坏，导致严重的酸中毒、体温过低和凝血病，也称为“致死三联症”。因此严重创伤应遵循以下原则——损伤控制性手术、控制性低血压、快速复温、限制晶体胶体输入、基于比例的血液复苏及治疗凝血病，来减少损伤、出血，以改善伤者预后[6]。

一般情况下，大约33％的血管损伤合并骨折，骨折是截肢的独立危险因素[7]。数字减影血管造影（DSA）是诊断血管损伤的金标准，多普勒血管超声和CTA血管造影仍是血管损伤最常用的无创检查[8]，有文献报道CTA对急性四肢动脉损伤诊断有很高的敏感性（98％）和特异性（99％）[9]。四肢血管损伤救治的要点在于迅速止血并恢复受伤肢体血流灌注，而其中的关键技术在于进行TIVS。对于严重多发创伤中的四肢主干血管损伤，一般认为损伤后6～8 h内是血管修复的治疗时限[10]，而实际大部分基层医院不具备相关血管修复经验，转诊至就近的创伤中心仍需要时间，因此血管转流是快速、有效的方法。

创伤性凝血病是由多因素引起的由凝血失调、纤维蛋白溶解改变、全身内皮功能障碍、创伤后炎症反应和血小板功能障碍引起的凝血功能紊乱[11]。因此，创伤的早期，建议传统的实验室测定方法，如PT/INR、纤维蛋白原水平和血小板计数和（或）黏弹性方法，对止血进行早期和反复监测，同时要早期纠正凝血功能紊乱、低体温及酸中毒[12]，体温过低会影响血小板功能改变、凝血因子功能受损、凝血酶抑制和纤维蛋白溶解。BE被描述为创伤早期评估和预后指标，通过其预测多发伤的休克、复苏需求、输血需求和死亡率[13]，对于严重创伤早期，建议予以氨甲环酸应用抗纤溶，建议在受伤后3 h内以1 g的负荷剂量输注超过10 min，然后在8 h内静脉输注1 g，建议初始补充3～4 g纤维蛋白原，这相当于15～20 U冷沉淀或3～4 g纤维蛋白原浓缩物，同时根据标准实验室凝血参数和（或）功能性凝血因子缺乏的黏弹性证据使用凝血因子凝血酶原复合物进行治疗[5]。

因此，对于严重创伤患者，排查活动性出血，早期确切止血，结合凝血指标及床旁边粘弹试验随时评估有无创伤性凝血病，早期打破凝血功能紊乱、低体温、酸中毒导致的“致死三联症”。一些钝性伤导致的皮下血肿或大血管损伤，存在隐匿性，应详细查体，完善检查，及时补充凝血底物及输注血制品，纠正凝血紊乱，发现血管损伤后，尽早确定动脉损伤部位和严重程度，早期行手术修复，同时观察肢体血液循环情况，积极处理骨筋膜室综合征及横纹肌溶解，根据脏器功能进行血液净化治疗，避免不良预后的发生。
